# The Book World of Medicine and Science

**Published:** 1901-01-12

**Authors:** 


### The Book World of Medicine and Science.

Food and the Principles of Dietetics. By Robert Hutchison, M.D.Edin., M.R.C.P. With plates and diagrams. (London: Edward Arnold. 1900. Price 16s. nett.)

It is seldom that we take up a book on dietetics which is at the same time so readable and so scientific as this is. The subject is a difficult one. From the scientific point of view it is full of uncertainty—the positive statements of the chemist requiring to be qualified at every turn by the less exact observation of the physiologist—while, on the other hand, from the popular writer's point of view, all is as plain as daylight, and the whole matter can be condensed into a series of aphorism. To steer a middle course between these two extremes, to give the complex facts as they exist, yet not to create confusion, to describe the pros and cons and yet to leave the reader something definite to take away with him, is by no means an easy task. It is one, however, which the author of the book before us has very successfully accomplished. The following example of the manner in which the lessons derived from scientific observation are turned to practical account will be of interest. Dealing with the question of the economic value of food he says: "In the market one pays for flavour and rarity, not for nutritive qualities. It is the demands of the palate which cost, not those of the stomach; and we pay for æsthetic qualities in foods just as in dress we pay for 'cut' and ornament, not for material to keep us warm. Suppose, for example, that one wants to buy a pound of fish. If sole is the form selected, it may cost 1s. 6d.; haddock would yield quite as much nourishment for 4d.; *i.e.*, in the former one is paying 4d. for nutriment and 1s. 2d. for other qualities. Or take the case of arrowroot. A pound of the best Bermuda costs 2s. 9d., St. Vincent only 3d. or 4d. Yet both are in a chemical sense merely starch, and physiologically their behaviour is identical. It is the same with cheese. A pound of Stilton costs 1s. 2d. The same amount of nutriment can be had in Dutch or American for 6d. These examples might be multiplied indefinitely, but they are sufficient to show that the maxim 'cheap and nasty' does not hold good for foods. . . . If one resolves to go in for luxury it is as well to do so knowingly, and not to imagine that one is nourishing the body when one is merely pleasing the palate." Again: "It is frequently our duty to see that the diet of a patient is enriched in special directions, most commonly perhaps in that of proteid or fat. There is no use in recommending to a poor man chicken and cream. He simply cannot afford to buy them. It is worth while to remember, however, that the cheapest sources of building material are *skim* milk, some form of fish (*e.g.*, herring or salt fish), cheese, the cheaper cuts of meat, and if his digestion be good the pulses, while the most economical forms of fat are margarine and dripping." These articles are within the reach of almost everybody, and, as the author says, in view of the fact that the working man must spend fully half his wages upon food, and that the poorer the man the larger must be the proportion so spent, "the pathetic thing is to find that it is just this class of the community whose food purchases are apt to be the most irrational. Surely there is room here for popular instruction."

The chemical constitution of foods and the physiology of digestion are very thoroughly considered, but the book is not devoted entirely to the scientific side of the question, fully as this is entered into. Scattered about its pages there is a very large amount of what for want of a better term we may call useful information by aid of which they are rendered interesting even to those who do not care for the more purely medical aspects of the problems dealt with. It is the author's intimate touch with the actualities of life which gives to this book much of its vivacity and lightens the load of the scientific facts which are found on every page, as instance the following passage, which comes at the end of the uses of sugar as a muscle food:—"It certainly seems as if it would be worth the while of captains of football teams to try the effect of serving round small cups of black coffee, strongly sweetened with glucose, at 'half-time,' instead of the usual lemon. They would probably be rewarded by the greater endurance of their men in the second half of the match."

The Sick and Wounded in South Africa: What I Saw and Said of Them and of the Army Medical System. By Mr. Burdett-Coutts, M.P. (Cassell and Co. 1900. Price 1s. 6d.)

The above is what is on the title page. On the cover we find that below the title there is subscribed the motto "Lest we forget." This motto no doubt exactly expresses the object of publishing this little book. It does not contain much that is new. It gives an outline of what the author did and where he went, the full text of his celebrated letter to the *Times* (Article No. IX. of "Our Wars and Our Wounded"), some of his speeches in the House of Commons, with extracts from the debates thereon; extracts from his evidence before the Hospitals Committee, an "Interview" on the nursing question reprinted from the "Hospital" Nursing Mirror, his election speech, and finally his suggestion for reform; and all along it gives a running comment which is written in a sufficiently lively and combative style to make the whole volume easy and interesting reading. Still, the motto is no doubt the reason for the book's appearance. Events have happened so quickly, the taking of Bloemfontein seems so far away, and the problems before us at the present are so embarrassing, that Mr. Burdett-Coutts did well in choosing the motto "Lest we forget." The proposals made for reform will require to be carefully considered, but we can hardly doubt that in one direction they contain the germ of what will in the future have to be done, namely, that the base hospitals should become practically civil hospitals, thus setting free the military doctors and male staffs for the performance of those duties at the front for which they have been specially trained. This, he says, contains the real germ of the reform of our army medical system in war time. "There must be a complete release to the civilian profession of the authority and management of base hospitals and of stationary hospitals which are strategically secure, in respect of all their functions which appertain to the treatment of the sick and wounded as such."

